# Supplementary material for: Chromosome structural anomalies due to aberrant spindle forces exerted at gene editing sites in meiosis
Source: J Cell Biol. 2018 Oct 1;217(10):3416–30. doi: 10.1083/jcb.201806072 (PMC6168266; doi:10.1083/jcb.201806072)
Supplement: Supplemental Materials (PDF) [file JCB_201806072_sm.pdf]

Supplemental material

Manil-Ségalen et al., https://doi.org/10.1083/jcb.201806072

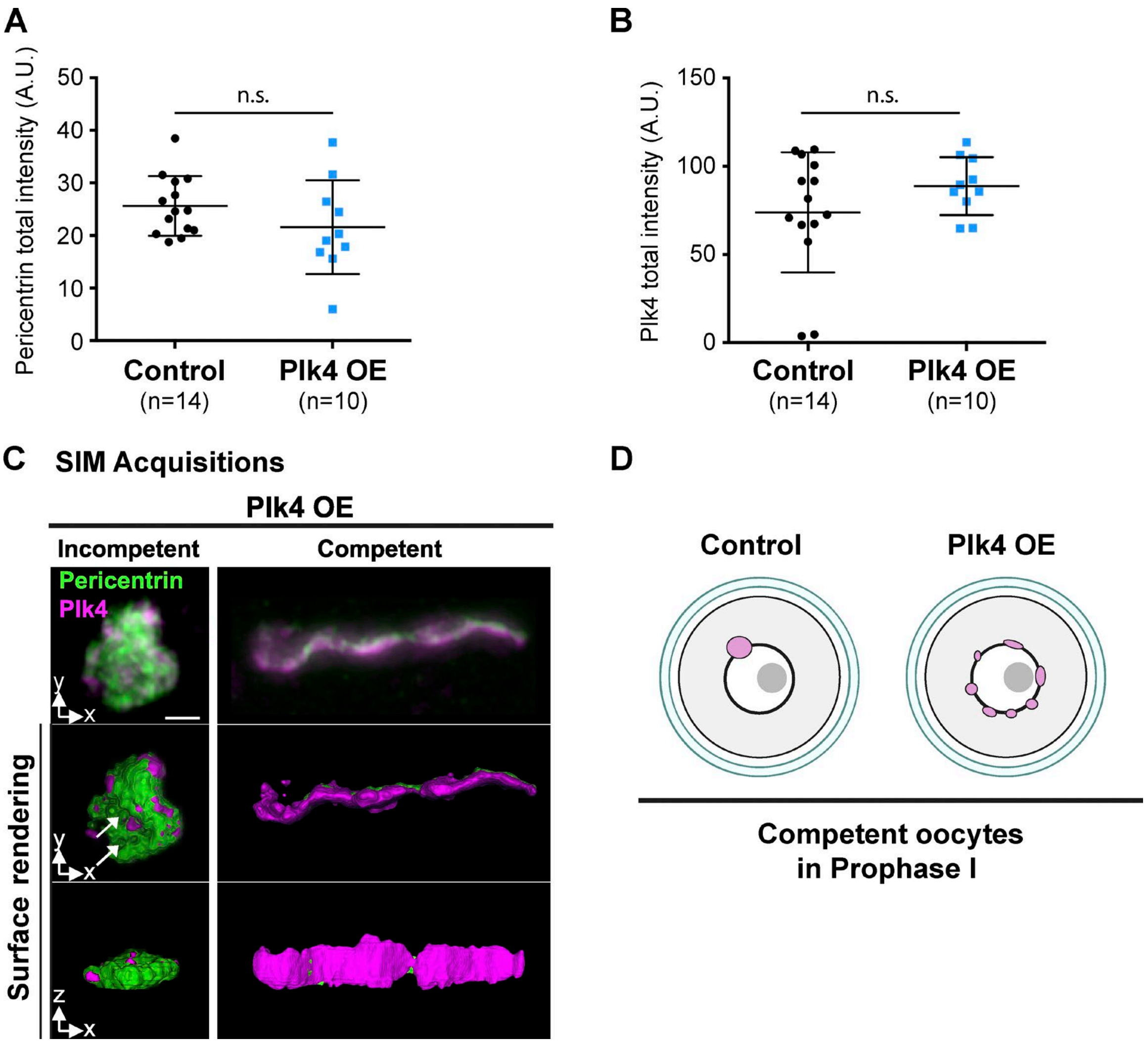

Figure S1. **Quantification of the pericentrin and Plk4 signals in the whole oocyte and SIM acquisition of aMTOCs in Plk4 OE oocytes. (A)** Measure of pericentrin signal intensity in the whole oocyte from control (black dots) and Plk4 OE (blue squares) as observed in Fig. 1 A; P = 0.2277. **(B)** Measure of Plk4 fluorescence intensity in the whole oocyte from control (black dots) and Plk4 OE (blue squares) as observed in Fig. 1 A; P = 0.1712. Statistical tests used are two-tailed *t* test with Welch correction; *n* is the number of oocytes. **(C)** SIM images of aMTOCs from incompetent (left) and competent (right) Plk4 OE oocytes observed in prophase I where Plk4 appears in pink and pericentrin in green. Upper panels correspond to one Z-section acquired with SIM. Middle and lower panels correspond to surface rendering views (X/Y views for middle and Z/X views for lower panels) of the aMTOCs reconstructed in 3D using Imaris. The white arrows point toward internal cavities within the pericentrin labeling in incompetent Plk4 OE oocytes. Scale bar is 1 µm. **(D)** Schematic representation of premature fragmentation of aMTOCs (pink) around the nucleus (in gray) in competent prophase I Plk4 OE oocytes. Error bars correspond to SD.

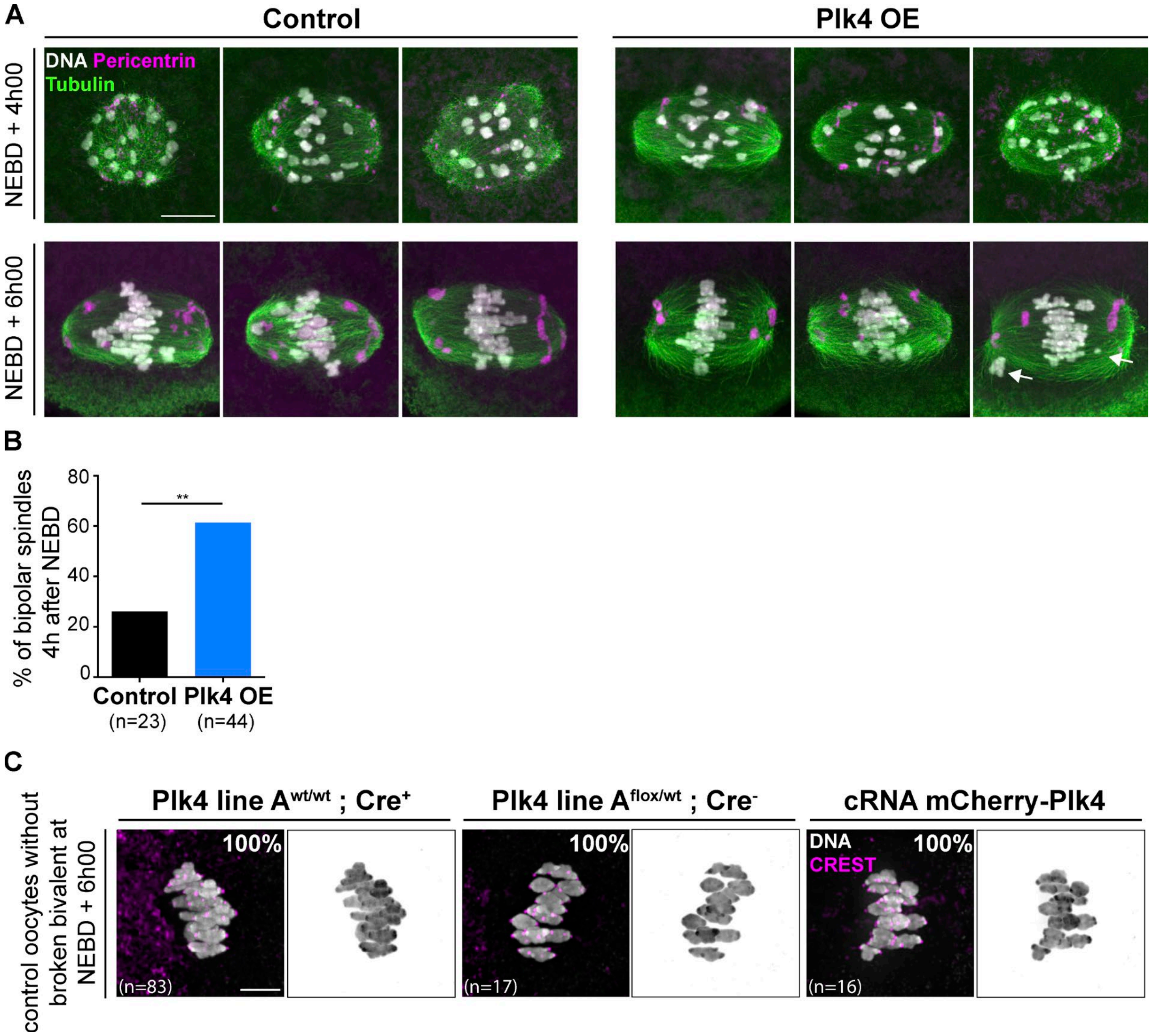

Figure S2. **Plk4OE oocytes present accelerated rates of spindle bipolarization as observed in fixed oocytes. (A)** Immunofluorescent staining of control (left) and Plk4 OE (right) oocytes fixed at NEBD + 4h00 and NEBD + 6h00. Tubulin is green, DNA is white, pericentrin is pink. The arrows show two pieces of a potentially broken bivalent. Scale bar is 10 µm. **(B)** Percentage of bipolar spindles in control (black histogram) and Plk4 OE (blue histogram) oocytes fixed at NEBD + 4h00; **, P = 0.0047 (Fisher's exact test); *n* is the number of oocytes. **(C)** Immunofluorescence images from control (Plk4 line A$^{wt/wt}$; Cre^{+} and Plk4 line A$^{flox/wt}$; Cre^{-}) and cRNA injected oocytes observed at NEBD + 6h00. DNA is in gray and CREST staining in magenta. The percentage of oocytes without any broken bivalent is indicated on the pictures; *n* is the number of oocytes.

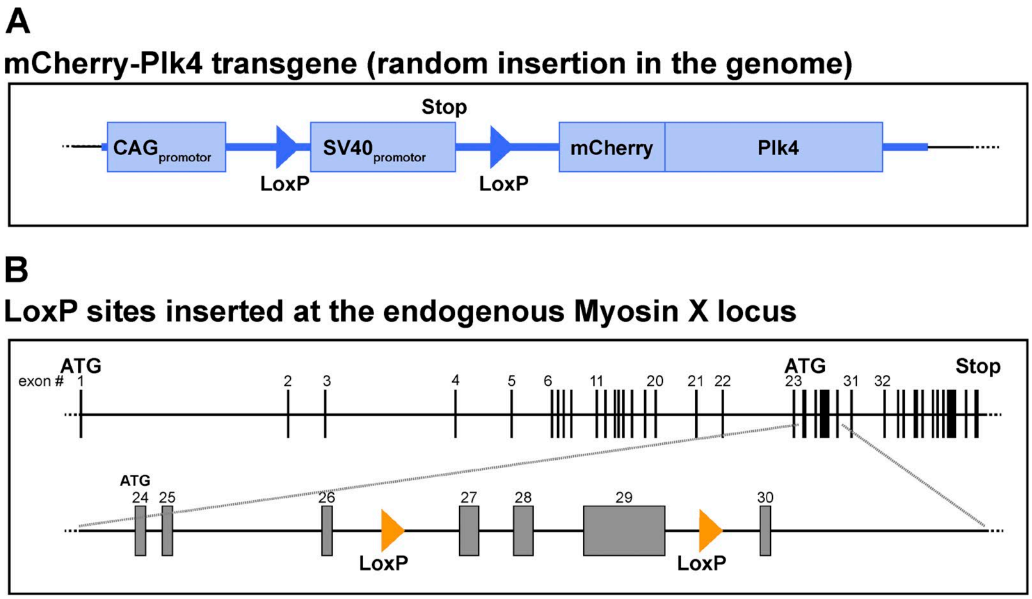

C

Line	Insertion site	% of oocytes with one broken bivalent
**Plk4 line A**	Chromosome 9<br>Plk4 Insertion A<br>chr9:43267673-43270267	**27.0%** (n=100)
**MyoX Flox**	Chromosome 15<br>LoxP sites in MyoX<br>chr15:25776791-25777552	**15.6%** (n=32)<br>typical break of the MyoX Flox Line only
**Plk4 line B**	Chromosome 12<br>Plk4 Insertion B<br>chr12:104996936-104997667	**10.2%** (n=68)

Figure S3. **Scheme illustrating the different genetic insertions used in the study.** **(A)** Scheme of the mCherry-Plk4 transgenic construction. **(B)** Scheme of the two Lox P insertions in the endogenous Myosin X locus. **(C)** Table illustrating genomic locations of three different Lox P insertion sites as well as the percentage of oocytes presenting one broken bivalent, probably at the insertion site schematized in the middle panel, coming from the respective genotypes Plk4 line $A^{flox/wt}$; Cre^{+}, Plk4 line $A^{flox/wt}$; Cre^{+}; $MyoX^{flox/wt}$, Plk4 line $B^{flox/wt}$; Cre^{+}; *n* is the number of oocytes.

https://doi.org/10.1083/jcb.201806072

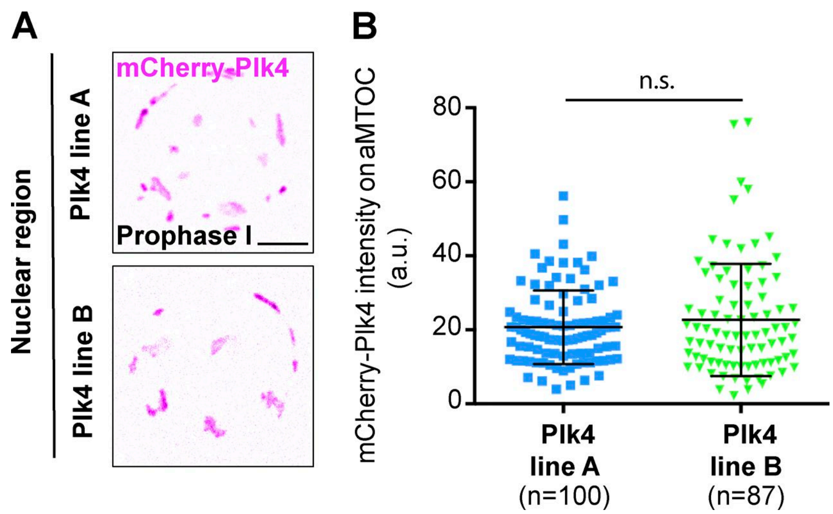

Figure S4. **Amount of mCherry-Plk4 on aMTOCs is comparable in line A and B in prophase I oocytes. (A)** mCherry-Plk4 signal (pink) around the nucleus observed in live prophase I of Plk4 OE oocytes from line A (upper image) and line B (lower image). Scale bar is 10 µm. **(B)** Quantification of the mCherry-Plk4 signal observed in A at aMTOCs; P = 0.986 (two-tailed Mann–Whitney); *n* is the number of oocytes. Error bars correspond to SD.

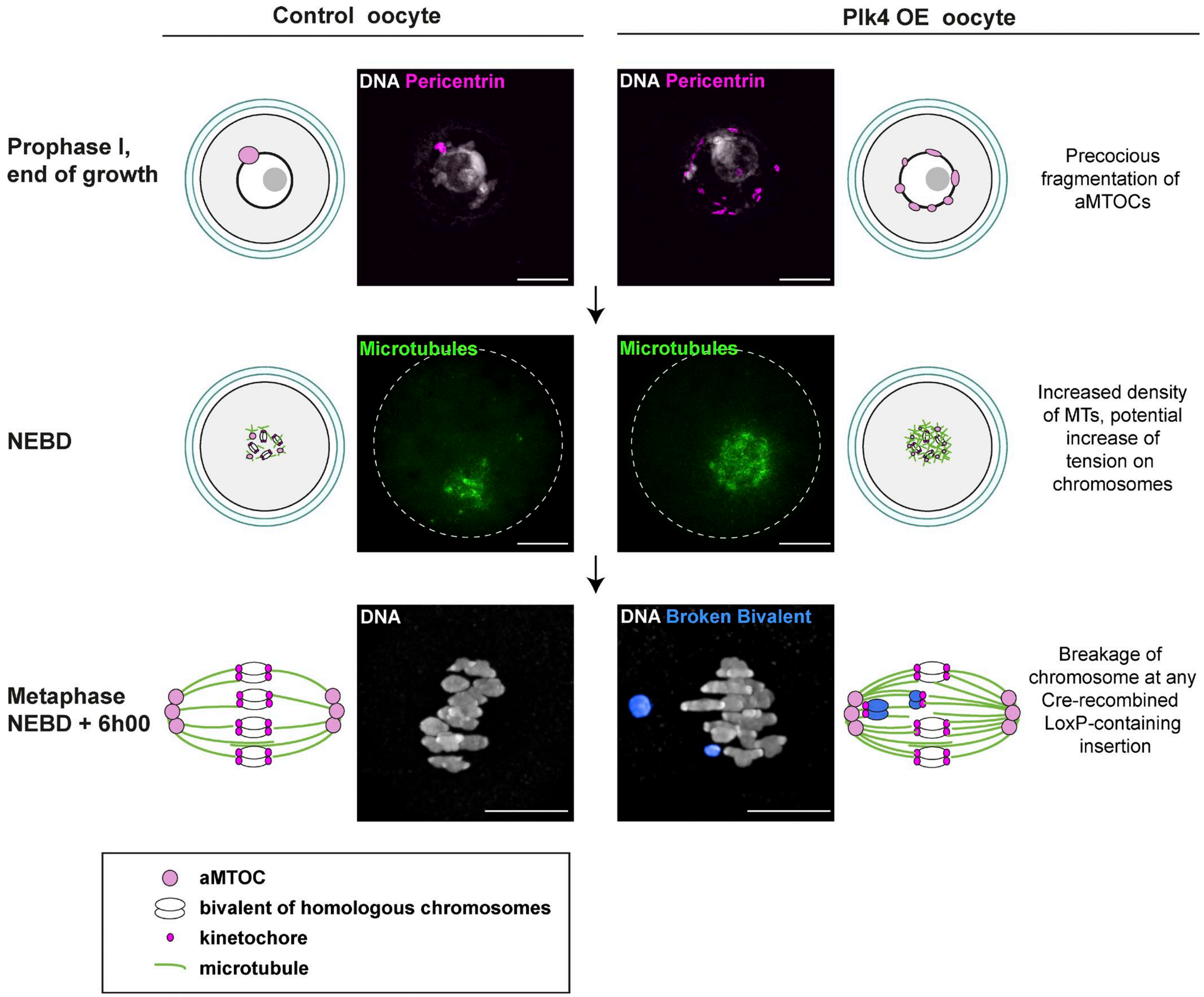

Figure S5. **Scheme recapitulating our model.** Our data are consistent with precocious fragmentation of aMTOCs in prophase I induced by Plk4 OE during oocyte growth. This in turn increases the density of MTs at NEBD, which promotes the breakage of one bivalent in any Cre-recombined Lox P–containing insertion during meiosis I. In the schemes, aMTOCs are in light pink, bivalents appear in white, MTs in green, kinetochores in dark pink. In the pictures, DNA appears white, pericentrin in pink, and MTs in green. The broken bivalent is highlighted in blue in both scheme and pictures.

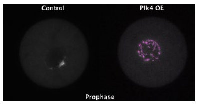

Video 1. **Spindle bipolarization is accelerated in oocytes overexpressing Plk4.** Early steps of spindle formation in control (left) versus Plk4 OE oocytes (right) expressing EB3-GFP. Oocytes were followed from prophase I exit until spindle bipolarization. EB3-GFP appears white and mCherry-Plk4 magenta. Timing (expressed in hours and minutes) is relative to NEBD. Video related to Fig. 2 D.

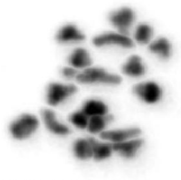

Video 2. **Chromosome fragment moving back and forth across the metaphase plate in Plk4OE oocyte.** Chromosomes labeled with Histone-GFP followed in Plk4 OE (line A) during meiosis I. Asterisks show the small chromosome fragment. Chromosomes appear in gray levels. Timing (expressed in hours and minutes) is relative to NEBD. Video related to Fig. 3 A.

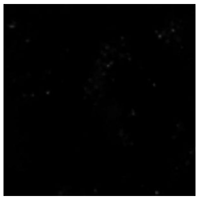

Video 3. **20 bivalents can be observed in control oocytes at NEBD + 6h00.** Z stack (Z-spacing: 200 µm) from a control oocyte observed at NEBD + 6h00, stained for DNA with DAPI (white) and kinetochores with CREST (magenta). Deconvolution of the stack of images has been performed as described in the Materials and methods section. Video related to Fig. S2 C.

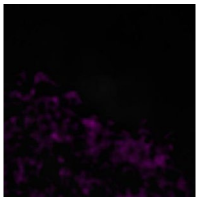

Video 4. **21 pieces of DNA can be counted in Plk4 OE line A oocytes at NEBD + 6h00.** Z stack (Z-spacing: 200 µm) from a Plk4 line $A^{flox/wt}$; Cre$^+$ oocyte observed at NEBD + 6h00, stained for DNA with DAPI (white) and kinetochores with CREST (magenta). Deconvolution of the stack of images has been performed as described in the Materials and methods section. Video related to Fig. 4 B.

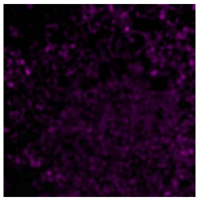

Video 5. **21 pieces of DNA can be counted in Plk4 OE line B oocytes at NEBD + 6h00.** Z stack (Z-spacing: 200 µm) from a Plk4 line $B^{flox/wt}$; Cre$^+$ oocyte observed at NEBD + 6h00, stained for DNA with DAPI (white) and kinetochores with CREST (magenta). Deconvolution of the stack of images has been performed as described in the Materials and methods section. Video related to Fig. 4 D.

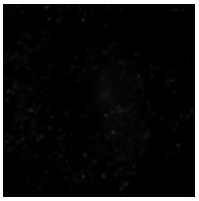

Video 6. **A new form of DNA break can be observed in Plk4 line $A^{flox/wt}$; Cre$^+$; MyoX$^{flox/wt}$ oocytes at NEBD + 6h00.** Z stack (Z-spacing: 200 µm) from a Plk4 line $A^{flox/wt}$; Cre$^+$; MyoX$^{flox/wt}$ oocyte observed at NEBD + 6h00, stained for DNA with DAPI (white) and kinetochores with CREST (magenta). Deconvolution of the stack of images has been performed as described in the Materials and methods section. Video related to Fig. 5 A.

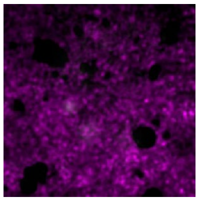

Video 7. **Bivalents can be easily counted in Plk4 OE oocytes observed at NEBD + 4h00.** Z stack (Z-spacing: 200 µm) from a Plk4 line $A^{flox/wt}$; Cre$^+$ oocyte observed at NEBD + 4h00, stained for DNA with DAPI (white) and kinetochores with CREST (magenta). Deconvolution of the stack of images has been performed as described in the Materials and methods section. Video related to Fig. 6 B.

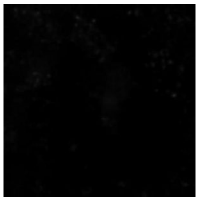

Video 8. **Bivalents can be easily counted in Plk4 OE oocytes treated with 50 nM nocodazole and observed at NEBD + 6h00.** Z stack (Z-spacing: 200 µm) from a Plk4 line $A^{flox/wt}$; Cre$^+$ oocyte treated with 50 nM nocodazole and observed at NEBD + 6h00, stained for DNA with DAPI (white) and kinetochores with CREST (magenta). Deconvolution of the stack of images has been performed as described in the Materials and methods section. Video related to Fig. 6 C.
